# Burnout, Psychological Capital and Health during COVID-19 Social Isolation: A Longitudinal Analysis

**DOI:** 10.3390/ijerph18031064

**Published:** 2021-01-25

**Authors:** Mariano Meseguer de Pedro, María Magdalena Fernández-Valera, Mariano García-Izquierdo, María Isabel Soler Sánchez

**Affiliations:** 1 Psychiatry and Social Psychology Department, Faculty of Psychology, Universidad de Murcia, 30100 Murcia, Spain; mgarciai@um.es (M.G.-I.); misoler@um.es (M.I.S.S.); 2 Psychiatry and Social Psychology Department, Faculty of Labor Sciences, Universidad de Murcia, 30100 Murcia, Spain; mariamagdalena.fernandez@um.es

**Keywords:** burnout, self-perceived health, psychological capital, COVID-19 social isolation

## Abstract

Background: Drawing on the impact of the COVID-19 global pandemic and its sanitary measures on coping strategies for preserving health, it is also necessary to add exposure to certain work stressors, such as burnout. The aim of the study was to assess the influence of the confinement situation caused by COVID-19 on the levels of self-perceived health and psychological capital in a sample of workers, as well as to analyze whether exposure to burnout before social isolation would help to explain the levels of health and psychological capital. Methods: Data were collected in a longitudinal design. Time 1 surveys (December 2019) were sent to a sample of 354 Spanish workers while in Time 2 (April 2020) the employees completed 235 questionnaires. Results: Our findings indicate a significant worsening of employees’ health perception (t = −4.13; *p* < 0.01) and psychological capital (4.10, *p* < 0.01) levels during mandatory confinement in Spain. Our results also revealed that emotional exhaustion is the only burnout dimension capable of explaining the variance of health while self-efficacy does regarding psychological capital. Conclusion: We conclude a significant reduction in self-perceived health and psychological capital during COVID-19 mandatory confinement, and that burnout acts as a predictor variable in both health and psychological capital variance.

## 1. Introduction

The 12 December 2019 marked, in the Chinese city of Wuhan, the appearance of a new human coronavirus with acute respiratory syndrome. Provisionally called 2019-nCoV or SARS-Cov2, it is the origin of a global pandemic, declared by the World Health Organization (WHO) on 11 March 2020. According to the latest data from the WHO [1], the case fatality rate (CFR; the ratio between confirmed deaths and confirmed cases) varies widely between countries. The case fatality rate has reached 4.9% in China, whereas in European countries differs from 0.5% in Iceland to 3.5% in Italy. In Spain, the case fatality rate had increased up to 12.2% in the worst moment of the pandemic. Nowadays, this rate is 2.5%. It is important to note that this information should be treated with caution due to the difficulties in collecting and interpreting the data. Currently, we finally can say there is a specific antiviral treatment. After the discovery of the vaccine against the virus, distribution to all countries has recently begun. In most of them, the vaccination process in the general population has already started.

Unfortunately, this pandemic not only has had effects on population health but also on the labor market. According to the latest International Labor Office report [2] in the third quarter of 2020 it has been estimated a decline in global working hours of 12.1%, equivalent to 345 million full-time jobs. In terms of work, younger and older people and especially women have been hit particularly hard by the COVID-19 economic crisis.

Considerable research attention has been directed toward physical and psychological effects caused by the COVID-19 virus on general population health and well-being [3,4,5]. Nevertheless, we consider a lack of research about how previous work stressors as burnout could increase the pandemic effects on employees’ health. Hence, the main goal of this study was, on the one hand, to examine the influence of the confinement situation caused by COVID-19 on employees’ self-perceived health and psychological capital levels. On the other hand, analyze whether exposure to burnout before social isolation would help to explain the levels of self-perceived health and psychological capital.

### 1.1. Social Isolation Measures’ Effects

One of the main strategies to contain the pandemic is confinement that is, legally forcing someone to stay in a certain place, usually the home. In Spain, for example, the confinement period has been prolonged for 97 days. During this time, it has been pointed out that due to the separation from loved ones, the feeling of loss of freedom and the uncertainty associated with the state of the disease, loss of job or professional status, this situation could have had dramatic long-term effects on general population psychological health [3,6]. Hence, these effects were already showed during the investigations carried out in the course of the previous epidemic associated with another SARS virus [7,8].

### 1.2. Psychological Capital as a Personal Resource

According to Hobfoll’s Conservation of Resources Theory [9] (p. 339), resources are defined as: “those objects, personal characteristics, conditions, or energies that are valued in their own right, or that are valued because of the act as conduits to the achievement of protection of valued resources”. The basic tenet of the Conservation of Resources Theory is that individuals strive to obtain, retain, protect, and foster those things that they have valued [9]. Hence, having a higher level of resources is favorable, especially in situations of high psychosocial stress [9].

Psychological capital is one of the resources that could be framed in Conservation of Resources Theory [10,11]. It has been proposed as a useful personal resource for coping with work stress [12,13], mobbing [14], burnout [15], or even unemployment [16,17]. Luthans, Youssef, and Avolio [18] propose psychological capital as a second-order construct that brings together four resources (optimism, resilience, hope, and self-efficacy), and is defined as a state of positive psychological development characterized by having the confidence to face challenges and difficult tasks (self-efficacy); make positive attributions about the present and future triumphs (optimism); visualize and persevere in the goals, as well as redirect the objectives when necessary to achieve success (hope); recover and even emerge stronger from adversity (resilience). The Conservation of Resources Theory by Hobfoll [9] defends that resources can be treated independently or integrated into more complex models. With this, the author pointed out the fact that some psychological concepts are better understood as the representation of a global multidimensional factor. That is, this theory defends the positive synergy between certain resources when they are part of a second-order construct that works better than each of its components separately [19]. Given the above, psychological capital research indicates that its four components have an underlying common bond that makes it a higher-order construct. This means that, if we take into account the four components of psychological capital as a whole, instead of focusing on them individually, their effects will have a greater impact than each of the four facets separately [18,19].

Even though psychological capital could help in high-stress situations, some authors have emphasized that the continued use of individual resources (tangible and intangible) to achieve vital objectives and face stressful situations, could result in resource deterioration [20]. When someone needs to face a high-caliber stressor, such as the confinement situation caused by COVID-19, two scenarios can be observed: (a) the resources are not adequate to cope with a certain stressor; (b) in certain situations, the stressor is so overwhelming that the resources available to a person, even if they are adequate, are not enough. In both cases, an irreparable deterioration of resources would occur [9]. Likewise, we consider that the confinement situation caused by COVID-19 is a serious stressor [3,4,5] before which, people’s psychological capital levels, could be deteriorated.

### 1.3. Burnout as a Work Stressor

Drawing on the impact of the COVID-19 global pandemic and its sanitary measures’ impact on health [3,4,5] and psychological capital [9], it is also necessary to add the exposure to certain work stressors, such as burnout [21].

Current literature supports the idea that burnout is a chronic work stressor, consisting of three symptoms: emotional exhaustion, cynicism, and low professional efficacy. Burnout syndrome is not an automatic process originated exclusively by work stressors, but the result of an interaction between personal characteristics and organizational features [22].

Many studies have reported a positive association between burnout syndrome and worsening of workers’ health [23], alcohol abuse consumption [24], sleep disorders [25], depression [26], sedentary lifestyle [27] or musculoskeletal pain [28]. Similarly, numerous investigations showed a negative association between burnout and psychological capital [15,29]. In line with this approach, psychological capital has clarified the relationship between burnout and job satisfaction [30], stress and burnout [31], as well as between work–family conflict and burnout [32].

### 1.4. Conservation of Resources Theory: Loss Cycle

For explaining the association between burnout syndrome, self-perceived health, and psychological capital during COVID-19 social isolation, we apply the loss cycle put forward in Hobfoll’s Conservation of Resources Theory [9]. This theory has been previously proposed as a valid theoretical framework to explain burnout outcomes [9]. As explained above, people strive to protect, conserve, promote, and obtain more resources [33]. In line with this argument, stressful situations appear when there is a real loss of resources or when they are threatened. Hence, when it is not possible to avoid the loss or recover from it, higher stress levels would give way to the loss cycle. This will, in turn, promote a predisposition towards the protection of the resources that are already owned but not always towards the creation of new resources [34].

The loss cycle hypothesis could be aligned with the idea that workers under high levels of stress perceive and create more work demands over time [35,36]. Apparently, this could be related to self-undermining behaviors, understood as certain behaviors that create various obstacles and end up undermining one’s performance [36]. Empirical evidence has shown that those workers who tend to self-weaken behaviors are more likely to experience higher levels of chronic stress, emotional demands, work pressure, and burnout [36,37]. Likewise, employees under self-undermining behaviors perceive a more stressful, confusing, and conflictive work environment, which ends up leading to an increase in labor demands [35,37]. All in all, this self-weakening conduct could be the fuel necessary to activate the loss cycle associated with burnout according to Hobfoll’s Conservation of Resources Theory [9]. Hence, those employees with higher levels of work stress are more likely to show self-debilitating behaviors, which will lead to higher levels of work demands and, consequently, will have fewer resources to deal with them, thus increasing their stress levels again [36]. That is, it is possible that the fact of being exposed to burnout before the start of the pandemic and its associated containment measures, makes workers fall into a loss cycle where they are not able to mobilize their resources to face this situation, thus affecting their health levels and weakening their levels of psychological capital [38]. Following Hobfoll [9] it is possible that when a worker has already seen deteriorated their personal psychological resources (in this case, psychological capital) trying to cope with burnout before confinement social isolation when they have to face another seriously stressful situation such as the one caused due to the COVID-19 virus, they experienced a lack of resources to deal with it, and therefore their health is affected. According to Hobfoll [9] when some resources have been invested in a previously stressful situation, they may not be available to meet future demands, leaving people exposed to the impact of stress.

According to the arguments exposed above, we hypothesize that:

**Hypothesis** **1.** *The confinement situation caused by the spread of the COVID-19 virus will hurt the perception of health.*

**Hypothesis** **2.** *The confinement situation due to the spread of the COVID-19 virus will harm psychological capital levels.*

**Hypothesis** **3.** *Previous COVID-19 confinement burnout workers’ level will be able to predict their psychological capital (a) and self-perceived health (b) during COVID-19 social isolation measures.*

## 2. Materials and Methods

### 2.1. Data Collection Procedure and Sample

A longitudinal non probabilistic design was followed. Data were collected in two-time points. Burnout, psychological capital, and health were assessed at Time 1 (December 2019), whereas psychological capital and health were also assessed four months later at Time 2 (April 2020), during the social isolation confinement caused by COVID-19 in Spain. The study was carried out under the recommendations of the ethics committee of Universidad de Murcia. All subjects gave written informed consent following the Declaration of Helsinki. Participants received an email with a direct link to the questionnaire hosted on the Universidad de Murcia inquiry platform to ensure confidentiality and anonymity. The email also contained the research goal, the instructions for the correct completion of the questionnaire, and a reminder of voluntary participation in the study.

Time 1 surveys were sent to a sample of 354 workers from different Spanish socioeconomic sectors. A total of 235 usable questionnaires were received, resulting in a response rate of 66.4%. About half of the respondent were men (59.1%; N = 139) with an average age of 36.62 years (SD = 13.43; range = 18–62 years). In terms of education, 37.8% (N = 89) had university studies, 26.8% (N = 63) followed vocational training studies, 18.7% (N = 44) had primary studies, and 16.6% (N = 39) had completed high school. Regarding job position, 35.3% (N = 83) held positions as a qualified worker, 21.7% (N = 51) were technicians, 18.3% (N = 43) worked as an unskilled worker, 12.8% (30) were a middle manager, and 9.4% (22) executives. Most respondents were permanent employees (53.2%; N = 125). Lastly, the average professional experience was 9.37 years (SD = 10.62, range = 1 month to 42 years).

Time 2 surveys were sent to a sample of 235 workers. A total of 198 usable questionnaires were received, resulting in a response rate of 84.3%. About half of the respondents were women (50.7%; N = 100) with an average age of 39.73 years (SD = 12.78; range = 18–58 years). In terms of education, 48.6% (N = 95) had university studies, 22.8% (N = 46) followed vocational training studies, 18.6% (N = 37) had completed high school, and 10% (N = 20) had primary studies. Regarding job position, 36.8% (N = 73) held positions as a qualified worker, 23.5% (N = 47) were technicians, 11.8% (N = 23) worked as an unskilled worker, 16.2% (32) were a middle manager, and 11.8% (23) executives. Most respondents were permanent employees (61.4%; N = 122). Lastly, the average professional experience was 11.74 years (SD = 10.57, range = 2 months to 38 years).

### 2.2. Measures

#### 2.2.1. Burnout

We assessed Burnout by the Maslach Burnout Inventory-General Survey (MBI-GS), developed by Maslach, Jackson, Leiter and Schaufeli [39] in the Spanish version by Salanova, Schaufeli, Llorens, Peiró, and Grau [40]. This scale is a 15-item self-report measure of job burnout that includes three dimensions, namely, emotional exhaustion, cynicism, and job self-efficacy. The items are rated from 1 (never) to 7 (every day). Some items are “I have become less enthusiastic about my work” (item 9) and “I have become more cynical about whether my work contributes anything” (item 13). Regarding internal consistency, Cronbach’s alpha coefficients were recorded for burnout at T1: emotional exhaustion α = 0.87; cynicism α = 0.82 and job self-efficacy α = 0.72. The test–retest reliability was r = 0.38 (*p* < 0.001) for the exhaustion subscale, r = 0.34 (*p* < 0.001) for the cynicism subscale, and r = 0.37 (*p* < 0.001) for the efficacy subscale.

#### 2.2.2. Psychological Capital

The OREA questionnaire developed by Meseguer-de Pedro, Soler, Fernández-Valera, and García-Izquierdo [41] was used to measure psychological capital. It consists of 12 items, three items for each psychological capital dimension. The OREA questionnaire items are rated on a four-point scale, that is, going from 1 (strongly disagree) to 4 (strongly agree). Sample items per dimension are: “In difficult times I usually expect the best” (Optimism); “I usually reach my goals even if there are obstacles” (Resilience); “I think my life is worth it” (Hope); “I am confident about I could effectively handle unexpected events” (Self-efficacy). The coefficient alpha for this scale was 0.82. Regarding internal consistency, the following internal consistency coefficients (Cronbach’s alpha) were found: α = 0.88 at T1 and α = 0.88 in T2; with a test–retest reliability of r = 0.60 (*p* < 0.001).

#### 2.2.3. Self-Perceived Health

As an indicator of the perception of psychological health, the GHQ-12 questionnaire by Goldberg and Williams [42] was used in the version validated by Rocha, Pérez, Rodríguez-Sánz, Borrel, and Obiols [43]. This instrument strives to estimate two kinds of phenomena: first the unfitness to experience and carry out activities in a functional, healthy way, and secondly the appearance of stressful phenomena. It contains 12 items referring to problems with well-being suffered in recent weeks (such as item 5: ‘Have you constantly felt overwhelmed and tense?’). It is evaluated via a four-point Likert scale ranging from 1 (‘not at all’) to 4 (‘much more than usual’), and therefore high scores indicate a lower level of health. Regarding internal consistency, the following internal consistency coefficients (Cronbach’s alpha) were found: α = 0.83 at T1 and α = 0.88 at T2, with a test–retest reliability of r = 0.60 (*p* < 0.001).

### 2.3. Data Analysis

The statistical analyses were performed using the IBM SPSS Statistics Versión 24.0 computer program. Bearing in mind the longitudinal design of the study, we proceeded as follows: first, internal consistency and reliability analyses (test–retest) were performed, followed by descriptive and bivariate correlations. Subsequently, mean comparisons were made between the two measurement moments to check if there were changes in the socioeconomic and demographic variables and to test the temporal persistence of the main study variables. Finally, for testing means differences, Student’s *t*-test was used. Finally, to analyze the longitudinal relationships, different multiple-step hierarchical regression analyses were carried out.

## 3. Results

We first inspected the descriptive statistics. Table 1 displays the means, standard deviations, and correlations of variables included in this study.

Secondly, we tested the existence of significant differences between self-perceived health and psychological capital in Time 1 and Time 2. For fulfilling this purpose, we applied the T Student proof. We found significant differences both in self-perceived health (t = −4.13; *p* < 0.01) and in psychological capital (t = 4.10, *p* < 0.01) between Time 1 and Time 2, which resulted in a confirmation of self-perceived health and psychological capital deterioration during home confinement. In a more detailed analysis, the items that contribute the most to a worsening of self-perceived health are: item 2 “has felt overwhelmed and stressed?” (t = −2.37, *p* = 0.02), item 10 “Have you been able to enjoy your daily activities?” (t = −3.70, *p* < 0.01), and item 12 “do you feel reasonably happy?” (t = −5.67, *p* < 0.01). Similarly, in psychological capital, significant differences were found in the four psychological capital dimensions: optimism (t = 2.88, *p* < 0.01), resilience (t = 3.47, *p* < 0.01), hope (t = 2.33, *p* < 0.05), and self-efficacy (4.37, *p* < 0.01).

Thirdly, we examined Time 1 predictors with the ability to commit to self-perceived health and psychological capital worsening in Time 2. To accomplish this goal, we carried out two hierarchical regression analyses (one for self-perceived health and another for psychological capital as output variables). We decided to introduce as input variables the three burnout dimensions (emotional exhaustion, cynicism, and self-efficacy) and age as a control variable, following the recommendations of previous health (Pinquart, 2001; Robert, 1999) and psychological capital research (Baron, Franklin & Hmieleski, 2013).

The results show that age and emotional exhaustion explain 27% of self-perceived health total variance in Time 2 (see Table 2). While, age and self-efficacy are responsible for explaining 24% psychological capital variance in Time 2 (see Table 3).

## 4. Discussion

The uncommon scenario caused by COVID-19 has meant a great change in the way of life of the population. It is not surprising the negative impact that such a situation can have on mental health in the short and long term. The present study examined the influence of the confinement situation caused by COVID-19 on the levels of self-perceived health and psychological capital in a sample of workers, as well as to analyze whether exposure to burnout before social isolation would help to explain the levels of health and psychological capital behind it.

Interestingly, the findings of this study provide support for the three proposed hypotheses: the first one stated that the confinement situation caused by the spread of the COVID-19 virus would have a negative influence on the perception of health; the second argued that the confinement situation caused by the spread of the COVID-19 virus would have a negative influence on psychological capital; finally, the third consisted of analyzing whether workers exposed to burnout before the confinement caused by COVID-19, would be more likely to enter a cycle of losses that would lead to a loss of their levels of self-perceived health and psychological capital.

Regarding the first hypothesis, our findings indicate a significant worsening of employees’ health perception during mandatory confinement in Spain. These results strengthen the research carried out about the psychological impact of extreme emergencies on health [44], as well as those that have specifically focused on the analysis of health and wellbeing of the general population during COVID-19 social isolation measures [3,4,5,6].

Respecting the second hypothesis, our results have also revealed a significant reduction in employees’ psychological capital levels during mandatory confinement. One possible explanation could be the idea of resource depletion framed in Hobfoll’s Conservation of Resources Theory [9]. According to previous research [20,45] the continued use of resources, such as psychological capital, in highly stressful situations, as could be the current one, can result in resources deterioration. In this regard, future research could try to elucidate whether the psychological capital decline is because perhaps its components (optimism, resilience, hope, and self-efficacy) are not the most appropriate to deal with this kind of substantial stressors, or that perhaps the stressor is so overwhelming that, although the resources are adequate, their levels are insufficient to cope with it.

Concerning the third hypothesis, our findings revealed, on the one hand, how age becomes a total variance predictor of both perceived health and psychological capital levels. What this seems to indicate is certain age-related changes affecting personal resources reserves would help to explain variations in physical health and psychological well-being [46]. To illustrate the relationship between psychological resources and age, one of the first studies carried out on this subject showed that as people aged, their resources were adapted to the demands they had to face [46]. Furthermore, from a cognitive perspective, it is considered that, as people get older, a greater need appears to allocate unused resources to compensate for the large number of resource losses that have occurred over the years. Therefore, although adults’ resource loss is practically inevitable, it is crucial to conserve and successfully mobilize the greatest number of resources to preserve health to a greater extent [45,46].

On the other hand, regarding burnout dimensions when explaining the variance of health and psychological capital levels, there is a significant and negative association between employees’ burnout in Time 1 with health and psychological capital in Time 2. These results corroborate previous research that has repeatedly shown the adverse burnout consequences on worker’s health [23,25,26], as well as those that have related burnout negatively and significantly to psychological capital [5,29,30,32]. It may be speculated that this is probably so because, applying the idea of loss resources cycle [9] together with the self-weakening concept (Bakker & Demerouti, 2017), those employees who perceive work-related stress are more likely to have self-debilitating behaviors, which will lead to higher levels of job demands and will have fewer resources to deal with these stressors, thus increasing their stress levels [36,37]. Hence, not being able to mobilize adequate resources to face the new situation, would significantly affect their health levels [9]. This argument could be illustrated with a situation in which a worker has invested his psychological resources (in this case, psychological capital) to cope with exposure to burnout before confinement so that when he has to face another serious stressor (in this case, mandatory social isolation measures) would not have sufficient levels of resources to deal with it.

Given the previous explanation, when analyzing our results in detail, it could be observed, first, that emotional exhaustion is the only burnout dimension capable of explaining the variance of health. In this regard, there is a consensus when it comes to affirming that emotional exhaustion is the central core of burnout, and as such the variable that would have the most influence on people’s health levels [47]. Traditionally, it has been thought that burnout (physical or mental) is a legitimate label that is used for problems that can occur both inside and outside the work environment since in any context people can feel exhausted. However, the other two remaining dimensions of burnout, cynicism, and professional inefficacy, would be reduced to the work context. Perhaps, in this case, emotional exhaustion is the only variable that contributes to explain the variance in health perception because, in addition to the argument exposed above, we are facing a non-work stressor (COVID-19 mandatory confinement). Second, the lack of professional efficacy is the only dimension of burnout that explains part of the total variance of psychological capital. If we understand job self-efficacy as a resource, following Ross and Mirowsky [48], when resources substitute for each other, the presence of one makes the absence of another less damaging. That is, the effect of having a specific resource is greater for those who have fewer alternative resources. In this case, workers with lower levels of psychological capital (alternative resource) may be more affected by the loss of a specific resource such as professional efficacy. Besides, following Youssef-Morgan and Luthans [49], and taking into account that one of the sources of self-efficacy (one of the components of psychological capital) is the experience of success in past situations, since one of the characteristics of burnout is the perception of professional ineffectiveness and therefore, the repeated feeling of failure when facing work tasks, this could prevent the necessary process for the attention, interpretation, and retention of positive and constructive memories, characteristic of psychological capital.

## 5. Theoretical and Practical Implications

Firstly, regarding the theoretical implications, the results of this study help to reinforce the application of the Conservation of Resources Theory for studying employee’s burnout and its consequent coping strategies. Secondly, two possible practical approaches emerge from this study: on the one hand, taking into account the substantial change in working conditions caused by COVID-19 and its influence on workers’ health, a jobs psychosocial risks reevaluation is needed both in face-to-face and teleworking modality. On the other hand, from the point of view of human resources management, it is necessary to build up employees’ personal psychological resources through training interventions, as well as to provide them with additional resources, such as social support from colleagues and supervisors.

## 6. Limitations

Several limitations of the present study should be acknowledged. First, the information was obtained through self-report questionnaires, which can produce response biases, exacerbating the common variance, and artificially increasing the correlations between variables [50].

Second, the presence of social desirability in the responses to the questionnaire could be another limitation. To try to detect the effects of this phenomenon on the results, it would be beneficial to include additional measurement scales that estimate its presence in subsequent studies [51]. An example is the Marlowe-Crowne Social Desirability Scale (MCSDS) [52].

## 7. Future Research

Further research is needed to focus on the prevalence of the disease in different settings. For example, future studies could examine if our results could differ according to the essential and nonessential workers distinction, between different labor market strata or even between labor layers.

## 8. Conclusions

Overall, the results of this study have shown a significant reduction in self-perceived health and psychological capital between the two periods analyzed (before and during the COVID-19 mandatory confinement). Specifically, burnout together with age act as predictor variables in both health and psychological capital variance between the two times included in this study. However, not all dimensions of burnout contribute to the same extent. Thus, regarding health effects, emotional exhaustion explains 27% of the total variance, while, for psychological capital, the dimension that contributes the most is job self-efficacy, explaining 24% of its variance.

## Figures and Tables

**Table 1:** Means, standard deviations, and correlations between the study variables.

	Mean	S.D.	1	2	3	4	5	6
1. Emotional exhaustion	13.21	7.75						
2. Cynicism	7.81	6.01	0.69 **					
3. Self-efficacy	30.38	4.19	−0.32 **	−0.36 **				
4. Psychological capital (T1)	41.57	4.94	−0.25 *	−0.18	0.61 **			
5. Self-perceived health (T1)	22.43	4.79	0.53 **	0.42 **	−0.45 **	−0.40 **		
6. Self-perceived health (T2)	24.90	6.07	0.48 **	0.38 *	−0.25 **	−0.24 *	0.60 **	
7. Psychological capital (T2)	39.01	6.46	−0.18	−0.14	0.48 **	0.61 **	−0.34 **	−0.27 *

T1: Time 1; T2: Time 2. * *p* < 0.05 (bilateral); ** *p* < 0.001 (bilateral). Note: Regarding self-perceived health, high scores indicate a lower level.

**Table 2:** Hierarchical regression analysis with self-perceived health as output variable.

Output Variable		Self-Perceived Health	
**Input Variables**	**β**	**R^2^**	**ΔR^2^**
Step 1			
Age	−0.37 **	0.12	0.12 **
Step 2			
Age	−0.27 *		
Emotional exhaustion	0.32 *		
Cynicism	0.11 (ns)		
Self-efficacy	−0.10 (ns)	0.27	0.18 **
Step 3			
Age	−0.28 *		
Emotional exhaustion	0.33 *		
Cynicism	0.12 (ns)		
Self-efficacy	−0.10 (ns)	0.27	0.00

* *p* < 0.05, ** *p* < 0.01.

**Table 3:** Hierarchical regression analysis with psychological capital as output variable.

Output Variable		Psychological Capital	
**Input Variables**	**β**	**R^2^**	**ΔR^2^**
Step 1			
Age	0.33 *	0.10	0.10 *
Step 2			
Age	0.24 *		
Emotional exhaustion	−0.03 (ns)		
Cynicism	−0.10 (ns)		
Self-efficacy	0.44 **	0.24	0.18 **
Step 3			
Age	0.23 *		
Emotional exhaustion	−0.01 (ns)		
Cynicism	−0.10 (ns)		
Self-efficacy	0.18 **	0.24	0.00

* *p* < 0.05, ** *p* < 0.01.

## Data Availability

The data presented in this study are available on request from the corresponding author. The data are not publicly available due to privacy restrictions.
